# The Potential of Microextraction Techniques for the Analysis of Bioactive Compounds in Food

**DOI:** 10.3389/fnut.2022.825519

**Published:** 2022-02-18

**Authors:** Jorge A. M. Pereira, Natalia Casado, Priscilla Porto-Figueira, José S. Câmara

**Affiliations:** ^1^CQM—Centro de Química da Madeira, Universidade da Madeira, Funchal, Portugal; ^2^Departamento de Tecnología Química y Ambiental, Escuela Superior de Ciencias Experimentales y Tecnología, Universidad Rey Juan Carlos, Madrid, Spain; ^3^Departamento de Química, Faculdade de Ciências Exatas e da Engenharia, Universidade da Madeira, Funchal, Portugal

**Keywords:** sample extraction, microextraction by packed sorbent (MEPS), solid-phase microextraction, food analysis, liquid chromatography (LC), gas chromatography, liquid-liquid microextraction (LLME), bioactive compounds

## Abstract

For a long time, the importance of sample preparation and extraction in the analytical performance of the most diverse methodologies have been neglected. Cumbersome techniques, involving high sample and solvent volumes have been gradually miniaturized from solid-phase and liquid-liquid extractions formats and microextractions approaches are becoming the standard in different fields of research. In this context, this review is devoted to the analysis of bioactive compounds in foods using different microextraction approaches reported in the literature since 2015. But microextraction also represents an opportunity to mitigate the environmental impact of organic solvents usage, as well as lab equipment. For this reason, in the recent literature, phenolics and alkaloids extraction from fruits, medicinal herbs, juices, and coffee using different miniaturized formats of solid-phase extraction and liquid-liquid microextraction are the most popular applications. However, more ambitious analytical limits are continuously being reported and emergent sorbents based on carbon nanotubes and magnetic nanoparticles will certainly contribute to this trend. Additionally, ionic liquids and deep eutectic solvents constitute already the most recent forefront of innovation, substituting organic solvents and further improving the current microextraction approaches.

## Introduction

Foods of plant-based origin (fruits, vegetables, legumes, whole grains, spices, and beverages) constitute a natural bioresource of phytochemical secondary metabolites—-polyphenols (flavonoids and non-flavonoids), vitamins, terpenes, carotenoids, capsaicinoids, glucosinolates, polyunsaturated fatty acids, among others. These metabolites exhibit powerful antioxidant (in scavenging free radicals that can easily react with nucleic acids, lipids, proteins and enzymes, causing tissue injury), anti-atherogenic, anti-inflammatory, antimicrobial, and antiproliferative properties, which protect us from degenerative diseases such as cancer (1, 2), aging (such as Alzheimer's and Parkinson's) (3), and cardiovascular disease (3, 4), longevity and assure good health (5). In addition, some of these bioactive metabolites are pigments (anthocyanins, flavones, carotenoids) which confer the typical color of foods and therefore can be isolated to be used as colorants in the food industry instead of the potentially harmful artificial colorants (butylated hydroxytoluene, tert-butyl hydroquinone, and butylated hydroxyanisole). In turn, the antioxidants are used to improve and increase the preservation, food shelf life, food quality and safety, and more recently as active ingredients in smart food packaging. Apart from the above mentioned field, secondary metabolites are also extensively being used in (i) pharmaceutical (antioxidant, anti-inflammatory and neuroprotective agents); (ii) cosmetic industry, as sun protectors, antiaging, anti-inflammatory and healing agents; and (iii) environmental applications as metal chelators, natural dyes (theaflavin) and biosorbents (anthocyanins- and tannins-based) (6). In recent years, scientific and industrial interest in bioactive compounds has increased considerably, supported by the growing and growing demand of consumers for foods that promote health and prevent certain diseases, namely those etiologically related to oxidative stress (7). These metabolites are not fundamental for the organism's vital processes, for their development and growth, but they can act as modulators of several important biological activities and functions giving them preventive properties of certain age-related pathologies and metabolic disorders.

*Polyphenols*—These secondary metabolites are notably recognized by their antioxidant activity. In plants, polyphenols stand out for their physiological and morphological importance, being used as a defense mechanism against oxidative stress, preventing damage to the DNA, protecting plants from UV radiation and aggression by parasites and predators (8). However, the enormous interest that these secondary metabolites have aroused in the international scientific community is related not only to their potential industrial applications but due to their beneficial effects on human health. Indeed, significant preventive potential in the development of chronic diseases mediated by oxidative stress including cardiovascular diseases (CVDs), cancer, type 2 diabetes mellitus, obesity, and neurodegenerative diseases (NDDs), is associated with polyphenols (8–10). According to chemical structure, polyphenols can be grouped into two categories: flavonoids and non-flavonoids. Flavonoids constitute the most important and abundant group, having a common structure of C6-C3-C6 diphenylpropanes consisting of 2 aromatic rings normally linked by an oxygenated heterocycle of 3 carbon atoms (11). This group of polyphenols can be subdivided into several subclasses represented by flavonols, flavones, flavanones, isoflavones, flavonoids and anthocyanins. The non-flavonoids group include as main subclasses phenolic acids, stilbenes, lignans and tannins.

Biogenetically, the synthesis of polyphenols involves a relatively complex network of secondary metabolic pathways that include the phenylpropanoid pathways through which phenolic acids are synthesized and the xiquimic acid pathway, where mainly flavonoids, tannins and stilbenes are formed.

The main precursors are the phenylalanine from the shikimate pathway and malonyl-CoA from the acetate pathway.

*Vitamins*—are found in a wide variety of fruits and vegetables, are reported as powerful dietary antioxidants with the potential to prevent heart, chronic inflammatory and neurodegenerative diseases (12). It has been shown that vitamin C could inhibit the oxidation of the complex formed between the low-density lipoproteins (LDLs) and proteins, contributing to reducing atherosclerosis, conserving the cardioprotective properties. Vitamin E prevents HDL from lipid oxidation and protects LDLs, DNA, and polyunsaturated fatty acids from oxidative damage. It, moreover, plays a role in the modulation of the immune response, hemoglobin biosynthesis, and stabilization of the structure of membranes.

*Carotenoids*—Located in subcellular organelles (plastids), mainly associated with proteins in the chloroplasts, carotenoids are naturally occurring pigments found in plants, fungi, algae, and bacteria, generally C40 tetraterpenes/tetraterpenoids formed from eight C5 isoprenoid units (13, 14). Consumption of carotenoids has been associated with a range of functions in human health, including a reduced risk of age-related macular degeneration and cataract (15). In addition to beneficial effects on eye health, by absorbing specific wavelengths of light, carotenoids also produce improvements in cognitive function and cardiovascular health, by blocking the formation and oxidation of LDLs, and may help to prevent some types of cancer, by limiting the abnormal growth of cells (16, 17). The most abundant carotenoid found in human serum is lycopene (carotenoid pigment responsible for the red color), found in high amounts in tomatoes, recognized as the most effective antioxidant among all the carotenoids.

*Glucosinolates*—are a large group of sulfur- and nitrogen-containing glucosides, which are abundant plant secondary metabolites, with nutritional effects and biologically active compounds found principally in the plant order Brassicales and related families of vegetables like broccoli, Brussels sprouts, and kale, which impart a characteristic pungent aroma and bitter taste. Therefore, they are frequently consumed as a normal part of the human diet (18, 19). Scientific studies support that consumption of glucosinolates, and their derivatives are associated with a preventive anticancer effect, namely of the lungs and alimentary tract, together with increased activity of detoxifying enzymes and resultant decreases in DNA damage (20).

### Bioactive Compounds in Disease Prevention

Plant-based foods rich in bioactive compounds could be used as functional ingredients for providing many health benefits. Several epidemiological studies related unhealthy dietary behaviors (malnutrition, tobacco, alcohol, physical inactivity) with chronic diseases such as cancer, type 2 diabetes, and cardiovascular diseases, which are leading causes of death in developed countries.

A diet rich in bioactive compounds, namely by the consumption of fruits, vegetables/legumes and spices, has a preventive action in the development of neurodegenerative diseases due to its ability to influence and modulate various cellular processes, such as signaling, proliferation, apoptosis, redox balance and differentiation (21, 22). It was demonstrated by Sarubbo et al. (23) that anthocyanins improve the function of hippocampal-dependent memory, preventing neurodegeneration by decreased levels of inflammatory biomarkers such as NF-kB, TNF-α, IL-1β, ROS, and RNS, reducing neuronal apoptosis through Bax, cytochrome c, caspase 3 and PARP-1 and increasing the levels of proteins such as Akt, GSK3β and BCL-2. Specifically, apigenin, one of the major polyphenolics in citrus fruit, parsley and celery, and quercetin, which is abundantly found in onions, green tea and apples, showed a potent anti-aggregating β-amyloid (Aβ) activity reducing the formation of amyloid plaques that are at the origin of the symptoms associated with Alzheimer's disease (23).

**Figure 1:**
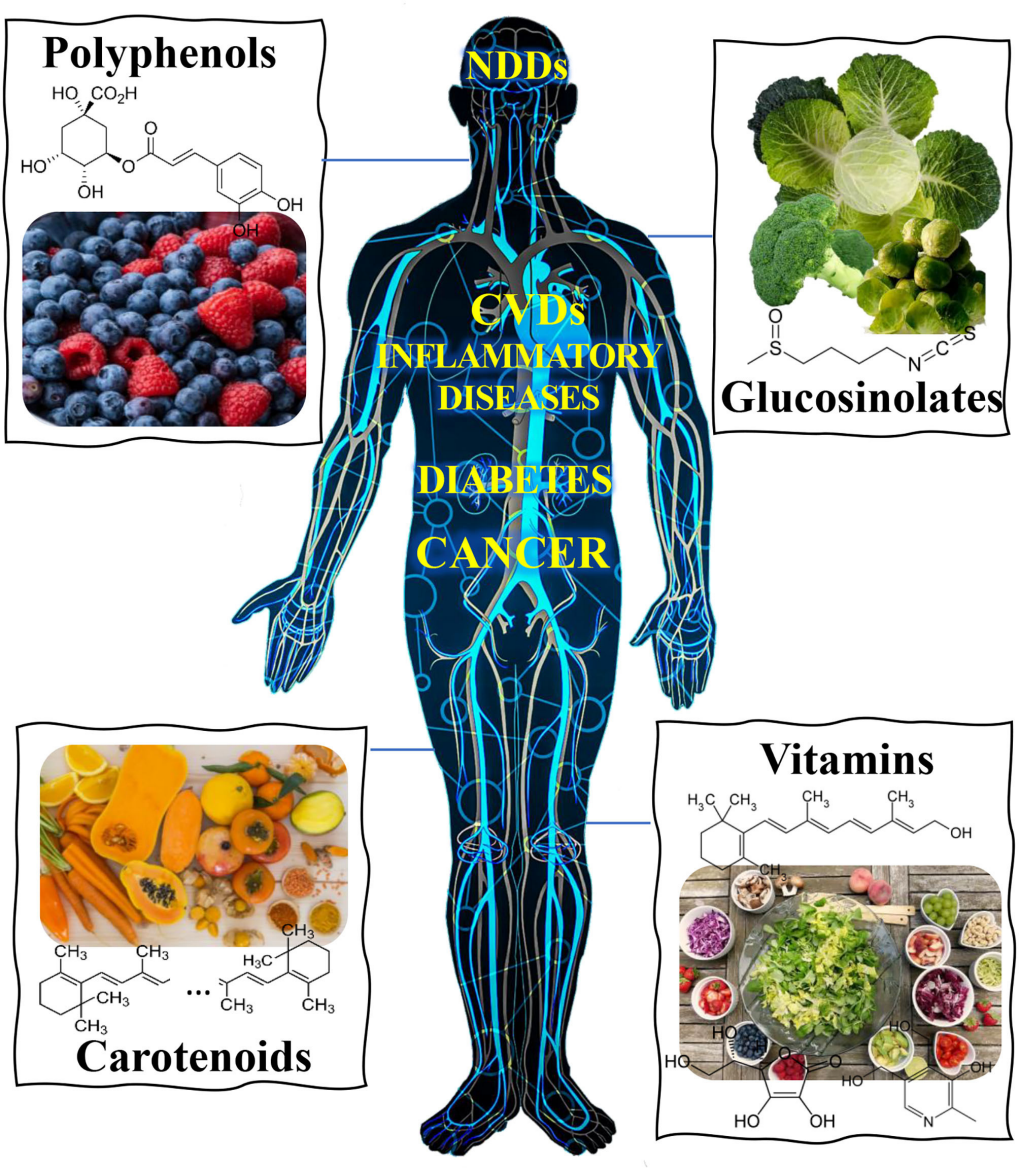
Overview of main classes of food bioactive compounds and reported activity against different human diseases, namely neurodegenerative (NDDs), cardiovascular (CVDs) and inflammatory diseases, cancer, diabetes, etc.

Moreover, some bioactive compounds can act in different phases of the cell cycle, repairing DNA damage and promoting cell apoptosis of the damaged cells to prevent carcinogenesis (24). Some bioactive compounds interfere with cancer initiation, promotion, and progression steps, modulating different enzymes and receptors in signal transduction pathways related to cell proliferation, differentiation, apoptosis, inflammation, angiogenesis, and metastasis. In addition, they also exert their preventive effect in decreasing resistance to multiple therapies and help regulate hormones and growth factors. They prevent DNA oxidative damage and modulate the immune system response (25). When combined with therapeutic agents such as Paclitaxel and Doxorubicin, some of the polyphenols allow for a decrease in the dose of the drug, thus decreasing the side effects of chemotherapy. The bioactive compounds are also associated with protective effects on the cardiovascular system (26). In addition to their antioxidant and anti-inflammatory activity, several mechanisms evidence these effects, namely those related to the lowering of blood pressure, the improvement of endothelial function, the inhibition of platelet aggregation and LDLs oxidation, also related to the prevention of the formation of atherosclerotic lesions, and with the enzymatic modulation expressed, for example, in the regulation of redox enzymes, thus contributing to reducing the risk of cardiovascular diseases. Flavonoids and catechins are the polyphenols that exert the most significant effect in reducing the risk of cardiovascular incidents. The flavonoids help to delay the development of atherosclerotic plaque and atherosclerosis by reducing endothelial dysfunction. In addition, flavonoids will interact with the membrane modifying its fluidity. Catechins help in smooth blood circulation since they can reduce the accumulation of cholesterol and its oxidation products in the walls of arteries. Moreover, they function as antiatherogenic agents by blocking oxidized LDLs-induced endothelial apoptosis. Figure 1 provides an simplified overview of the main classes of food bioactive compounds and targeted diseases. Several *in vitro* and *in vivo* assays have been developed to evaluate the antioxidant activity of several plant-based foods rich in bioactive compounds (27, 28). These assays have demonstrated the importance of secondary metabolites such as phenols, flavonoids, carotenes, among others, with verified antioxidant activity for the prevention of degenerative disorders. The most common *in vitro* assays include 2,2-diphenyl-1-picrylhydrazyl radical (DPPH) assay; total radical trapping antioxidant parameter (TRAP) assay; oxygen radical absorbance capacity assay (ORAC); reducing power assay; ferric reducing-antioxidant power (FRAP); superoxide radical scavenging activity, determination of phenol content by the Folin–Ciocalteu method; and total flavonoid content. The *in vivo* assays include total flavonoid content; reduced glutathione (GSH) assay; glutathione peroxidase (GPx) assay; glutathione-S-transferase (GSt) assay; glutathione reductase (GR) assay; and superoxide dismutase (SOD) assay. A huge amount of high-resolution analytical methodologies, from chromatographic techniques (29, 30) to nanobiosensors (31, 32) constitute powerful analytical tools that might be applied in food analysis, able to cope with a large number of analytes in food matrices, including those responsible for bioactive potential. The selection of the analytical technique depends mainly on the chemical nature, properties and required robustness and sensitivity of the target analytes to be determined.

## Samples Extraction in Food Analysis

### Importance of Sample Extraction in Food Samples

**Figure 2:**
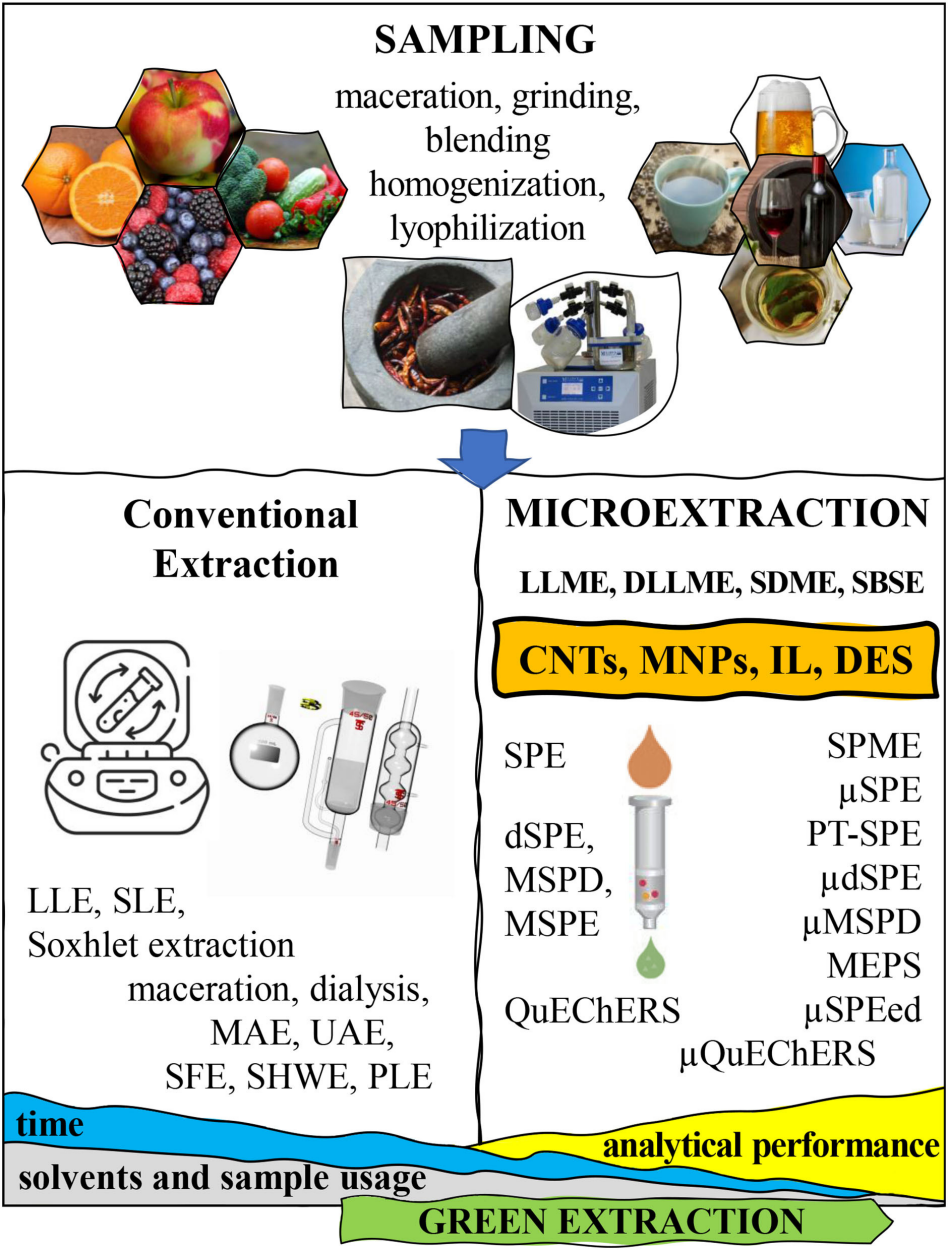
Overview of the most relevant steps in the extraction layout of food samples, from sampling to microextraction of the target analytes.

Food samples are extremely complex matrices, as they present a wide variety of compounds in their composition, such as proteins, fats, sugars, salts, vitamins, additives, etc., with different polarity, size and chemical properties. For this reason, the determination of a specific bioactive compound in a food sample is not an easy task, since its extraction and detection can be hampered due to the interferences caused by the rest of the components present in the food matrix (33). These matrix interferences usually have a negative impact on important analytical parameters, such as the limits of detection and quantification, the accuracy, and the precision, leading to low sensitivity and selectivity in the analysis (34). Consequently, food samples, in general, cannot be analyzed directly. Instead, they must undergo a proper extraction procedure before the instrumental analysis. Therefore, in food analysis, it is of utmost importance to carry out a sample extraction that enables clean-up, selective extraction, purification and even pre-concentration of the target analytes, to facilitate and enhance their following instrumental analysis (35) (Figure 2). Indeed, sample extraction is one of the most critical steps of any sample preparation technique. The main aim of sample extraction is to minimize the sample complexity and eliminate most matrix interferences. This can be obtained through the isolation, purification and pre-concentration of the analytes of interest in a matrix, while simultaneously turning them into a form compatible with the instrument used for their subsequent analysis. This step often involves a chromatographic technique (e.g., high performance liquid chromatography – HPLC, or gas chromatography, GC) coupled to a powerful detector (normally mass spectrometry (MS) or ultraviolet (UV) detectors) (36). The extraction of a specific analyte depends on several parameters, like its solubility, the mass transfer between the sample and the solvent, and the matrix interferences. Moreover, the nature of the sample also plays an important role. In this sense, liquid food samples can be directly extracted with a suitable solvent. However, solid food samples require a previous preparation step before extraction to improve the efficiency of the process, which involves reduction of the sample particle size (grinding) for better mass transfer and, normally, a drying step to decrease the moisture (as it may reduce the extraction efficiency). Often freeze drying, or lyophilization, is applied to solid samples before grinding, particularly when they present high levels of water content. These procedures are also important to homogenize the sample and ensure that each aliquot is representative of the whole sample composition, therefore avoiding biased results of food composition in the analyte of interest. As we will discuss below, as extraction approaches are evolving to sophisticated microextraction procedures requiring progressively lower sample amounts, sample homogenization is becoming critical to obtain reproducible results. After these operations, the solid powder can be extracted with a suitable solvent (solid-liquid extraction, SLE) obtaining a sample extract solution that can further be treated as a liquid sample (37) (Figure 2).

### Conventional Sample Extraction Approaches

Solvent-based extraction techniques, such as liquid-liquid extraction (LLE), Soxhlet extraction, maceration, membrane extraction (dialysis), microwave-assisted extraction (MAE), ultrasound-assisted extraction (UAE), supercritical fluid extraction (SFE), super-heated water extraction (SHWE) and pressurized liquid extraction (PLE), have been traditionally applied to the extraction of bioactive compounds from food samples (37–39). These techniques are based on the contact of the sample (liquid or solid) with a suitable solvent. However, these solvent-based techniques do not achieve selective extraction of the analytes, besides providing insufficient clean-up of the samples. For these reasons, in the last decades, the combination of solvent-based procedures with sorbent-based extraction techniques has aroused special interest in the extraction of food samples, due to the higher extraction capacity and selectivity achieved (40). The sorbent-based extraction techniques are based on sorption processes that enable the selective extraction of the analytes from the matrix by their retention in a sorbent material with specific and related characteristics, allowing the purification and concentration of the analytes at the same time. Accordingly, in the extraction of food samples, there is usually a first step involving the contact of the sample with an organic solvent under different conditions (pressurized, assisted, etc.) to obtain a sample extract, which is afterwards purified in the next step by a suitable sorbent-based extraction technique. In the case of liquid samples, they can be directly purified with the sorbent material. In this context, solid-phase extraction (SPE) is the main sorbent-based extraction technique and has become the extraction procedure of choice in the food field, replacing the classic LLE, due to its selectivity and versatility (37). Moreover, the great variety of sorbent materials available to perform sorbent-based extractions (e.g., reversed-phase, ion-exchange, mixed-mode, molecularly imprinted polymers, magnetic nanoparticles (MNPs), multiwalled carbon nanotubes (MWCNTs), metal-organic frameworks, etc.) offers a wide range of possibilities to achieve selective extraction of analytes with different chemical properties. This has opened in the last years new research lines focused on the development of new sorbent materials designed with specific characteristics depending on the type of analytes, so a better extraction efficiency and suitable clean-up can be achieved (33–36, 40–42).

### Different Solvent- and Sorbent-Based Formats for the Extraction of Food Samples

For many years, the conventional extraction techniques LLE and SPE have been popular choices for the extraction of food samples. However, LLE presents some inherent drawbacks, such as large volumes of organic solvents, limited ability to extract polar compounds, long time consumption, etc. Consequently, SPE is preferred in food analysis, as it provides better extraction efficiency and requires less consumption of organic solvents than LLE (43). Nevertheless, since the first appearance of the SPE, other sorbent-based procedures based on the SPE extraction principle have been proposed over the last decades, such as dispersive SPE (dSPE), matrix solid-phase dispersion (MSPD) and magnetic SPE (M-SPE). The difference between the SPE and these techniques lies basically in how the sorption process between the analytes and the sorbent is carried out. For instance, in SPE and MSPD the sorbent material is packed in an extraction cartridge, whereas in dSPE and MSPE the sorbent is dispersed in the sample solution. In the case of MSPD, the sorbent is previously dispersed and blended with the sample, and then the mixture is packed in the cartridge, while in SPE only the sorbent is packed in the cartridge. On the other hand, any type of sorbent can be used in dSPE, but in MSPE, only MNPs can be employed. In general, these sorbent-based techniques arose to improve SPE, by reducing the number of steps, decreasing the volume of organic sorbents, enhancing the sorbent-analyte interaction, and making easier the extraction procedure (33, 41, 42).

## Emergent Microextraction Approaches for Food Analysis

The current trend in sample extraction is to perform sustainable procedures that fulfill the Green Analytical Chemistry (GAC) requirements, which mainly involve using minimal amounts of solvents and samples, carrying out fewer steps in the procedures and reducing the waste generation (43, 44). This way, more cost-effective and environmentally friendly extraction procedures can be achieved. This can be accomplished by the miniaturization of the extraction procedures. Accordingly, the conventional sorbent-based extraction procedures have been scaled down and applied to the extraction of bioactive compounds in food samples. For instance, the miniaturization of SPE has been carried out for the extraction of flavanones from citrus fruits (45), miniaturized dSPE has been applied to the extraction of p-coumaric acid and p-hydroxybenzoic acid from fruit juices (46), miniaturized MSPD was used to extract different polyphenols from olive fruits (47) and flavonoids have recently been extracted from different food items by miniaturized dSPE (48). Likewise, the QuEChERS (Quick, Easy, Cheap, Effective, Rugged and Safe) strategy first developed in 2003 (49), which involves simultaneous extraction and clean-up of samples, has also been successfully miniaturized and applied to the extraction of 12 polyphenols in baby foods (50). Nonetheless, in the past decades, several microextraction methods based on SPE and LLE have emerged with new configurations and formats intending to improve the drawbacks of conventional extraction procedures and address some key challenges, such as the need to use lower sample volumes and perform quicker analysis. In this sense, LLE has been the axis to develop different solvent-based microextraction methods, including liquid-liquid microextraction (LLME), dispersive liquid-liquid microextraction (DLLME), single-drop microextraction (SDME), liquid-liquid-liquid microextraction (LLLME) and hollow-fiber liquid-phase microextraction (HF-LPME). On the other hand, sorbent-based extraction techniques have evolved beyond the SPE principle, leading to the design and development of more innovative extraction formats and devices, such as solid-phase microextraction (SPME), stir-bar sorptive extraction (SBSE), microextraction by packed sorbent (MEPS), μSPEed^®^ and pipette-tip SPE (PT-SPE). These microextraction techniques are increasingly being proposed as an alternative to conventional extraction procedures in food analysis, as they enable “greener” approaches that meet the GAC requirements. Figure 2 offers an overview of the context of microextraction as an evolution from sampling to emergent microextraction approaches. To organize their discussion, selected examples of the analysis of bioactive compounds in foods involving microextraction were primarily divided according to the physical state of the sorbent used in the retention of the target analytes. This results, in a simplistic way, in microextraction approaches using solid sorbents, like exposed solid sorbents (SPME), packed sorbent particles (μSPE, pipette-tips SPE, MEPS, μSPEed), or dispersed sorbent particles (QuEChERS, dSPE, MSPD), described in more details in Table 1. In turn, Table 2 is devoted to applications involving a liquid sorbent, like different formats of miniaturized LLE (μLLME, μDLLME, μSDME). The use of ionic liquids (ILs) and deep eutectic solvents (DES) as extraction solvents is being successfully applied to different formats of microextraction and will be also discussed.

**Table 1:** Selected examples of microextraction of bioactive compounds using solid sorbents.

**Extraction format/methodology**	**Target analytes/sample**	**Method description/analytical performance (LOD)**	**References**
HS-SPME	VOCs/mangoes	Blended 5.0 g juice + 1.5 g of NaCl in 15 mL capped vial, 80 rpm stirring; 10 min-equilibration; 30 min DVB/Carboxen/PDMS extraction (40°C); GC desorption	(51)
HS-SPME	VOCs/tangerines	5 mL tangerine juice or 250 mg peels (20 mL HS glass vial, magnetic stirring); NaCl 10% (w/v); HS-SPME extraction (40 min, 40°C); GC-MS analysis	(52)
EPT-SPME/PT-SPME	Alkaloids, flavonoids/TCM	**Sample pretreatment**: liquid samples (1.0 mL) centrifugation (5,000 g, 10 min, RT); **EPT-SPME**: hollow polypropylene fiber attached to 1,000 μL pipette tip; 10 mg sorbent + 2 mg sodium bicarbonate placed in the tip; 500 μL sample supernatant + 100 μL sodium dihydrogen phosphate (0.5 mol L^−1^) loaded into the tip; 1.2 min dwelling; sample withdrawn; washing (200 μL water); desorption (100 μL MeOH-ammonia (90:10, v/v) withdrawn into the tip; 1.5 min dwelling; solvent withdraw; UHPLC-UV analysis (20 μL). **PT-SPME**: 10 mg sorbent placed in the tip; 500 μL sample supernatant + 100 μL phosphate buffer (0.5 mol L^−1^, pH 6.5) loaded into the tip; 1.2 min dwelling; 200 μL water washing; desorption: 100 μL MeOH: ammonia (90:10, v/v) withdrawn into the tip; 1.5 min dwelling; UHPLC analysis/1.02–2.98 μ gL^−1^	(53)
HS-SPME	Trans-resveratrol/wines, grape juices	30 min extraction (1.5-mL amber vials; HS-SPME with magnetic stirring; 6.6% EtOH + 10% NaCl); 15 min fiber soaking (mobile phase); desorption; LC-UV analysis/0.4 ng mL^−1^	(54)
MMF-SPME	Phenolic acids/fruit juices	MeOH + water activation; 20 mL sample (25 mL vial + stirring bar, 8 × 2 mm); MMF direct immersion in sample solution; 40 min, low stirring; MeOH/1% AA aqueous (v/v, $\frac{1}{4}$ 90/10) desorption (400 μL, 20 min)/0.17–0.57 μg L^−1^	(55)
NTME	VOCs/lemon	250 mg sample (20 ml HS glass vial), equilibration (10 min, 50°C); 30 mL gas phase (30 cycles) loaded through the NTD (pre-attached to 1 mL syringe); GC-MS analysis	(56)
PT-SPE	Flavonoids/TCM	100 μL sample loaded into tip (5 μL s^−1^ flow rate); 1.0 min dwelling; 500 μL water washing; 100 μL ACN desorption (5 min dwelling); HPLC analysis (20 μL). **SPE**: 500 μL sample loaded (positive pressure) onto preconditioned Sep-Pac C18-360 mg cartridge (3.0 mL MeOH + 3.0 mL water); 2 × 5 mL water washing; 3.0 mL ACN elution; evaporation to dryness (40°C, N_2_ stream); 100 μL ACN reconstitution, vortex, and centrifugation (10,000 rpm, 10 min); HPLC-UV analysis (2 μL)/0.027–0.045 μg mL^−1^	(57)
M-MWCNTs-SPE	GA/pomegranate rind	5 g dry pomegranate powder + 50 mL EtOH (20%, v/v); filtration; pomegranate rind extract (20 mL) + M-MWCNTs@GA-MIPs (30 mg) incubation (oscillator, 60 min); collect M-MCNTs@GA-MIPs with captured GA (magnet); GA desorption (EtOH-HAc (95:5, v/v); evaporation to dryness (N_2_ stream); MeOH reconstitution (5 mL); HPLC-UV analysis/0.001 μg mL^−1^	(58)
M-MIP-SPE	Chlorogenic acid/ fruit juices	30 mL fruit juice + 60 mg Fe_3_O_4_@CGA-MIPs; 30 min, RT; Fe_3_O_4_@CGA-MIPs separated magnetically, EtOH–HAc (95:5, v/v) elution (6 h); filtration (0.22 μm nylon membrane); HPLC-UV analysis/0.01 μg mL^−1^	(59)
M-SPE	Flavonoids/ tea, wine	2 mL samples into a centrifuge tube; 8 mg magnetic graphene composites (GO/Fe_3_O_4_); 2 mL phosphate buffered solution (0.02 M); sonication (5 min), vortex (15 min in an oscillator); remove supernatant while holding GO/Fe_3_O_4_ composites with a magnet; target analytes elution (2 × 1 mL acetone containing 1 % HAc); evaporate to dryness (60 °C; N_2_ stream); MeOH reconstitution (0.5 mL); filtration (0.45 μm); HPLC analysis (10 μL)/0.2-6.0 ng mL^−1^	(60)
MISM-PT	GA/juices	1 mL MeOH + 1 mL water washing; 6.0 mL orange juice sample loaded on MISM-PT (0.15 mL min^−1^); 150 μL hexane washing; 250 μL gallic acid elution; extract concentrated to dryness (N_2_ stream); dissolved in 50 μL mobile phase; HPLC-UV analysis (20 μL)/7.0 μg L^−1^	(61)
PT-SPE	Estradiol/milk	MIOMS-ir (3 mg) packed into 100 μL pipette tip (cotton capped at both ends); preconditioning (1 mL MeOH + 0.5 mL water); 0.5 mL milk sample loading; 0.3 mL water washing; 0.5 mL ACN-acetic acid (96:4, v/v) elution; evaporation to dryness (N_2_ by gentle nitrogen blowing); residue redissolving in the mobile phase (0.5 mL) for LC-FLD analysis/6.00 ng L^−1^	(62)
SBA-15-μSPE	Flavanones/citrus fruits	Citrus edible parts blended; extract filtration; packed 25 mg SBA-15 (1-mL cartridge); preconditioning (1 mL MeOH, 1 mL water); diluted juice (20 μL + 4.97 mL water, pH 7) loading (1 mL min^−1^ flow rate); washing (1 mL water); vacuum elution (1 mL MeOH); dryness (block heater); resuspension (100 μL MeOH); UHPLC analysis/4.26 ng mL^−1^	(45)
SBA-15-μSPE	Triterpenoid saponins/TCM	0.25 g dried powder + 70% EtOH (25 mL); US (ice bath, 30 min); filtration, dilution to 50 mL. **SPE**: packed 25 mg SBA-15 (1-mL cartridge); preconditioning (1 mL MeOH, 1 mL water); diluted extract loading (1 mL min^−1^ flow rate); washing (1 mL water); vacuum elution (1 mL EtOH 70%); dryness (N_2_ stream); resuspension (200 μL MeOH); UHPLC-CAD analysis/**0**.461-0.976 μg mL^−1^	(63)
Ni/Co-NO_3_-LDH-μSPE	Phenolic acids/fruit juices	2D ultrathin Ni/Co-NO_3_ layered double hydroxide nanosheet (6.0 mg) added to 5.0 mL sample solution (pH = 7.0); 6 min US; centrifugation at 3,000 rpm for 5.0 min; supernatant decantation; sorbent dissolution in 75 μL TFA (8%, v/v) under US (1.0 min); HPLC analysis (20 μL)/0.1 μg L^−1^	(46)
MEPS	Furanic derivatives/sugarcane honey	eVol^®^ syringe + RCX sorbent (4 mg); conditioning (250 μL MEOH + 250 μL FA 0.1%); 500 μL sample loading (3 withdraw cycles); 100 μL MeOH washing; 100 μL MeOH: FA 0.1% (95:5, v/v) elution + 100 μL FA 0.1% elution; UHPLC-PDA analysis/30.6-737.7 μg kg^−1^	(64)
MIP-MEPS	Caffeine/soft & energy drinks	4 mg MIP; 150 μL sample (pH 4); 200 μL MEOH: acetic acid (9: 1, v/v) elution, one draw-eject cycle/1 μg mL^−1^	(65)
MEPS, μSPEed	Polyphenols/baby food	**MEPS**: eVol^®^ syringe (500 μL) + 4 mg DVB/HLB sorbent BIN; 100 μL MeOH + 100 μL FA 0.1% conditioning; 250 μL (pH 2.0) sample loading (10 withdraw cycles, flow rate 20 μL s^−1^); 100 μL MeOH: FA 0.1% (95:5, v/v) elution; HPLC-PDA analysis. **μSPEed**: digiVol^®^ syringe + PS/DVB-RP sorbent. 100 μL MeOH + 100 μL FA 0.1% conditioning; 100 μL (pH 2) sample loading (10 withdraw cycles); 50 μL MeOH: FA 0.1% (95:5, v/v) elution; UHPLC-PDA analysis/1.37, 13.57 μg kg^−1^	(66)
μSPEed	Phenolics/tea	eVol^®^ syringe (200 μL/min flow rate) + PS/DVB-RP sorbent; conditioning (500 μL MeOH + 200 μL FA 0.1%); 2 × 100 μL sample loading; 50 μL MeOH: FA 0.1% (95:5, v/v) elution; UHPLC-PDA analysis (2 μL)/3.5-16.8 ng mL^−1^	(67)
SE/dSPE	Polyphenols/juice, smoothies	50 mg of HMS-C18 conditioned with 1 mL MeOH/water (50:50, v/v); 10 min stirring (300 rpm); add 5 mL sample extract, 20 min stirring (300 rpm); filtration (0.45 μm); elution (2 × 3 mL MeOH/water 95:5, v/v pH 2); evaporation to dryness; MeOH resuspension (500 μL); UHPLC-MS analysis/0.01-1.7 μg mL^−1^	(68)
CNF-NLPNE	Phytohormones, ginsenosides/standard solutions	**CNFs/CFs (standard solutions)**: 1- Conditioning step: US (5 min) of 3000 CNFs/CFs (1 cm length, 10.00 mg) in NCS; 2- solvent loading step: CNFs/CFs soaking NCS (100 μL, 3 s); 3- extraction step: CNFs/CFs dipped in 1 mL (unstirred, stirred, or vortexed solutions); 4- desorption step: CNFs/CFs dipped in NCS (1 mL); US (30 s). **Needle-tip device procedure**: 1- solvent loading: CNFs/CFs wetted in acidified ACN (10 μL NCS); 2- sampling: needle inserted into the sample; 3- extraction: 25 μL sample juice drawn from fruit and discarded (2.5 μL s^−1^); 4- Desorption step: 10 μL acidified ACN (FA 1%) drawn (2.5 μL s^−1^ flow rate); LC-MS/MS analysis/0.01-5.53 ng mL^−1^	(69)
M-DES-dμSPE/M-dμSPE	Morin, quercetin, kaempferol/foods	Sampling: apple juice (commercial juice extractor) centrifuged (5,000 × g, 15 min), supernatant collected; green tea infusion (0.5 g/100 mL boiling water, 10 min) cooled to RT; dried onion (50°C, 7 h) extraction (1 g/10 mL HCl 25%, 80°C, 30 min); centrifugation (5,000 × g, 10 min), 10 × dilution double distilled deionized water. **M-DES-dSPE**: 150 μ DES3 + 7 mg of SiO_2_@Fe_3_O_4_ MNPs; US (1 min); 10 mL sample (1 mL glass syringe) + solution, equilibrium in few seconds; magnet to isolate MNPs; elution (250 μL EtOH, 2 min US). HPLC-UV analysis (25 μL). **M-dSPE**: like DES-dμSPE but not using DES/0.2–2.98 μg L^−1^	(48, 70)
MSPDM	Phenols/olive fruits	Glass mortar blending (60 s) powdered sample (50 mg) + 25 mg middle-molecular-weight chitosan (dispersant); column packing, plunger compression; elution (3 × 0.5 mL MeOH (60%, v/v); evaporation to dryness; MeOH redissolution (200 μL); centrifugation (5 min, 13,000 rpm), UHPLC-Q-TOF/MS analysis (2 μL)/69.6-358 ng g^**−1**^	(47)
Graphene nanoplatelets-MSPDM	Phenolic acids/plant preparations	Graphene nanoplatelets and sample mixture (mortar) packed into 1-mL SPE cartridge; 0.2 mL elution (different solvents); dilution (10 ×, elution solvent); centrifugation (5 min, 13,000 rpm); UHPLC-ECD analysis (2 μL)/1.19–4.62 ng mL^−1^	(71)
ILs-MSPDM	Neohesperidin, naringin/lime fruit	50 mg sample powder + 150 mg Florisil dispersant placed into glass mortar and homogenized (1 min); transfer to SPE cartridge (3 mL) with frits in both ends; [Bmin]BF_4_ (0.4 mL, 250 mM) elution under vacuum (28.0 bar); centrifugation (13,000 rpm, 5 min); UHPLC analysis/4.08, 5.04 μg g^−1^	(72)
ILs-MSPDM	GA, resveratrol, etc/TCM	Grinding (25 mg sample + 25 mg disorganized silica, 2 min, mortar); SPE packing; 1 mL 150 mM 1-dodecyl-3-methylimidazolium bromide elution; 10 min 14,000 rpm centrifugation; filtration; 2 μL UHPLC analysis	(73)
MSPDM	Coumarins, phenolic acids/TCM plants	Grinding (25 mg sample + 25 mg Diol, 3 min, mortar); SPE packing; elution (MeOH 70%); centrifugation (14,000 rpm, 10 min); UHPLC analysis/0.08–0.12 μg mL^−1^	(74)
MSPDM	Narirutin, naringin, hesperidin, neohesperidin/citrus fruit	30 mg dispersant + 25 mg sample; 1 min grinding (agate mortar); homogenization; 2.5 mL MeOH, 5 min vortex; eluent diluted 20 × 5 min at 13,000 rpm centrifugation; IM-QTOF-MS analysis/3.70–6.52 ng mL^−1^	(75)
MOF-MSPDM	Saponins/ginseng leaves	MOF-808 grinding (25 mg leaves, 30 s); glass cartridge packing; 200 μL MeOH elution/0.087-0.114 μg mL^−1^	(76)
QuEChERS	Trigonelline, caffeine, chlorogenic acid/green coffee beans	0.200 g powdered coffee beans; 10 mL 1% acetic acid; 90 min US (RT); 1,700 g centrifugation; decantation; 50 μL coffee extract diluted to 4 ml (1% acetic acid); 4 mL ACN; mix; 0.5 g NaCl + 1.0 g MgSO_4_; vigorous shaking; centrifugation; ACN phase (upper) analyzed by UV-VIS (50 μl diluted to 4 ml); aqueous phase (lower) extracted twice with ACN; UV-VIS analysis/1.51, 0.960, 0.879 mg L^−1^	(77)
μQuEChERS	Polyphenols/baby food	0.3 g sample (2 mL tube) + 0.2 g μQuEChERS mixture (buffered salts, 4:1:1:0.5) + acidified (0.1% FA) ACN:EtAc (1 mL, 1:1, v/v); vortex (10 s) + US extraction (5 min); centrifugation (5 min, 5,000 rpm); 700 μL supernatant transferred to 2 mL PTFE dSPE tube (75 mg MgSO_4_ + 12.5 mg PSA); vortex (30 s); centrifuge (5 min, 4,000 rpm); filtration (500 μL extract, 0.22 μm PTFE filter); evaporation to dryness; MeOH reconstitution (100 μL); UHPLC-PDA analysis/0.04–0.46 μg g^−1^	(50)
SALLE	Naringenin/fruit juices	5 mL samples + 1.7 mL ACN; 30 s vortex; 1 g (NH_4_)_2_SO_4_, 5 min shaken; 5 min 9,000 rpm centrifugation; 500 μL upper phase evaporated; 500 μL MeOH reconstitution; HPLC analysis/0.1 μg mL^−1^	(78)
SALLE	Isoflavones/soy milk	2.5 g samples (pH 6) + NaCl + ACN; 300 rpm shaking; 5 min 3,000 rpm centrifugation (25 ± 4°C); ACN phase filtration; UHPLC–MS/MS analysis/1–30 pg	(79)
SALLE	Matrine alkaloids/TCM	200 μL sample + 8 mL 10% NaCl (pH 12); 120 μL CHCl_3_ injected rapidly; 30 s mix, 3 min rest; CHCl^3^ volatilized naturally; MeOH reconstitution; 20 μL HPLC analysis/0.06, 0.08 ng mL^−1^	(80)

*ACN, acetonitrile; BIN, barrel insert needle; [Bmin]BF_4_, 1-butyl-3-methylimidazolium tetrafluoroborate; CNFs, carbon nanofibers; CNTs, carbon nanotubes; DES, deep eutectic solvents; dSPE, dispersive solid-phase extraction; DVB, divinylbenzene; ECD, electrochemical detection; EPT-SPME, effervescent pipette tip solid phase microextraction; EtOH, ethanol; FA, formic acid; FGO-TD-PTFE, functional graphene oxide thermal desorption poly(tetrafluoroethylene); FLD, fluorescence detector; GA, gallic acid; GC-MS, gas chromatography-mass spectrometry; HAc, acetic acid; HLB, hydrophilic-lipophilic-balanced; HS, headspace; ILs, ionic liquids; LDH, layered double hydroxide; M-SPE, magnetic solid-phase extraction; MIP, molecular imprinted polymer; MIOMS-ir, molecularly imprinted ordered mesoporous silica imprint-removed silica; MISM, molecularly imprinted silica monolithic; ME, microextraction; MeOH, methanol; MEPS, microextraction by packed sorbent; MMF, multiple monolithic fiber; MNPs, magnetic nanoparticles; MOF, Metal–organic framework; MSPDM, matrix solid-phase dispersion microextraction; MWCNTs, Multi-walled CNTs; NCS, nanoconfined solvent; NTD, needle trap device; NTME, needle-trap microextraction; PDA, photodiode array; PDMS, polydimethylsiloxane; PS/DVB-RP, reverse phase polystyrene-divinylbenzene sorbent; PT- pipette tip; RAMIPs, restricted access molecularly imprinted polymers; RT, room temperature; SALLE, salting-out liquid-liquid extraction; SBA-15, Santa Barbara Amorphous 15 molecular sieve; SPME, solid-phase microextraction; TCM, traditional Chinese medicine; US, ultrasonication; VOCs, volatile organic compounds; UHPLC, ultrahigh performance liquid chromatography; UV, ultraviolet analysis; μSPE, micro solid-phase extraction*.

**Table 2:** Selected examples of microextraction of bioactive compounds in liquid sorbents.

**Extraction format/methodology**	**Target analytes/sample**	**Method description/LOD**	**References**
LLME	Caffeine/tea, coffee	20 mg tea leaves powder + 20 mL EtOH/10 mL water (beaker, pH adjusted to 2.5); stirring (40 min, 45°C); supernatant decantated and filtered (0.45 μm); 1.0 mL extracted sample in 2:1 EtOH/water mixture (conical glass test tube); 150 μL DCM rapidly injected to form three solvents mixture; 20 s high speed vortex; add 300 μL water; vigorous shake, rest to obtain two phases; caffeine sedimented (bottom organic phase of DCM); centrifugation (5,000 rpm, 4 min)/0.05 μg mL^−1^	(81)
VA-LLME	Phenolic acids/honey, iced tea, coffee drinks	Sample solution (pH 1.5–1.8, 10 mL volumetric flask); propyl acetate: pentanol: hexanol (1:2:1.5, v/v/v, 400 μL) vortex extraction (45 s, 2,500 rpm); rest 1 min; transfer top layer (~200 μL) to centrifuge tube (0.5 mL) containing 40 μL KOH (0.02 M); vortex (60 s, 2,500 rpm); collect aqueous fraction (bottom layer); HPLC analysis/0.05–0.68 μg L^−1^	(82)
VA-LLME	Hydrophilic phenols/olive oil	5 mL oil samples diluted to 5 mL (hexane) + 100 μL 1 M HCl; 2 min vortex; 10 min 4,000 rpm centrifugation; 40 μL acidic aqueous phase (lower phase) analyzed by differential pulse voltammetry (DPV) using SPCEs/0.022 mg L^−1^	(83)
SHS-LLME/DLLME	Piperine/pepper	Sample grinding; 0.10 g + 5.0 ml ACN 45% (v/v); 1 min vortex; 2 min 6,000 rpm; filtration; 3.0 mL saturated NaCl (Salting-out extraction – SOE); 1 min vortex; 2 min 6,000 rpm; 50 μL SOE diluted to 4.0 mL (deionized water); 1.5 mL SHS + 1.0 mL 20 M NaOH, 10 s vortex; recover switched-off SHS layer; dilute 50% prior HPLC-DAD analysis/0.2-0.6, 0.7–2.0 μg mg^−1^	(84)
LLME (US and salt-assisted)	Oleuropein/olive oil	0.01 g + 10 mL extraction solvents mixture (phosphate buffer, with variable pH, ACN, and THF); US (25°C); 5 min 4,000 rpm centrifugation; 1 mL liquid phase + NaCl (salting out); 10 μL organic phase HPLC analysis/0.5 μg mL^−1^	(85)
Ball mill-assisted DES-based extraction	Tanshinones/Salvia miltiorrhiza Bunge	Oven-dried, sliced, and crushed samples (0.05 g) mixed with DES (1.0 mL) in 2.0 mL Lysing Matrix D tubes (1.4 mm ceramic spheres, 1.1 g); centrifugation; LC-MS analysis	(86)
DES-LLME	Caffeine/soft drinks	1 mL sample; 50 μL THF (aprotic solvent); 50 μL DES (choline chloride–phenol); HPLC-UV analysis/0.03 μg mL^−1^	(87)
DES-LLME	Quercetin/wine, foods	Ground dried (5 g) and liquid (3 mL) samples + 10 mL MeOH; 40 min US extraction (RT); filtration/6.1 μg L^−1^	(88)
DES-LLME	Rare ginsenosides/Kang'ai injection	8 mL sample + 400 μL DES in glass test tube; add 150 mg inorganic salt; shake to obtain homogeneous solution; add 400 μL THF (turbidity is observed); add 150 mg Fe_3_O_4_; inject N_2_ (to obtain homogenous turbid droplets and make the magnetic nanoparticles absorb the droplets simultaneously); collect nanoparticles (magnet); washed thoroughly (little amount EtOH); concentrate to 200 μL; filtration (0.22 μm PTFE); HPLC analysis/10.2–137.8 ng mL^−1^	(89)
DES-LLME	Curcumin/food, herbal tea	Curcumin standards + phosphate buffer (2 mL, pH 4) in 50 mL centrifuge tube; 400 μL DES (ChCl: Phenol, 1:4) as water-miscible extraction solvent injected rapidly into solution to form homogeneous solution; THF (400 μL, emulsifier) injected into solution; cloudy solution (formation of insoluble self-aggregates) was obtained; US (2 min) to homogenize DES droplets in the aqueous phase (curcumin extraction); centrifugation (4,500 rpm, 5 min); discard top water phase; DES rich-phase containing curcumin completed to 1 mL with EtOH; curcumin concentration determined by UV (425 nm)/2.86 μg L^−1^	(90)
DES-DLLME	Phenylpropanoids/vegetable oils	2 mL oil samples + 2 mL *n*-hexane; 45 μL PhAA/TMG in 200 μL isopropanol (dispersing solvent) rapidly added; 10 s vortex; 5,000 g centrifugation (5 min); 5 μL DES solution UHPLC analysis/0.07–0.01 μg mL^−1^	(91)
DLLME (OS vs. DES)	Phytosterols/cow milk	2.0 mL milk sample; 1.0 or 1.25 mL ACN (OS-DLLME or DES-DLLME, respectively); 30 s vortex; 4 min 4,000 rpm centrifugation; supernatant phase (0.8 mL) + 70 μL CCl_4_, mix; 3 min 4,000 rpm centrifugation; bottom phase evaporated (N_2_ stream); ACN (95%, v/v) reconstitution; HPLC-UV analysis/0.3–0.9, 0.09–0.32 ng mL^−1^	(92)
NADES-LLME	Polyphenols/Greek medicinal plants	Pulverized material (0.1 g) + NADES (80% v/v, 10 mL), manual vigorous shaking; US extraction (80°C, 90 min, 140 w); centrifugation (15,000 rpm, 10 min); dilution 1:20 distilled water; spectrometric analysis	(93)
NADES-LLME	Anthocyanins/Catharanthus roseus	Samples (50 mg) + NADES (1.5 ml); stirred (40°C, 30 min); centrifugation (1,300 rpm, 20 min), filtration (0.45 μm filter); dilution (1:2, 3%FA); HPLC-DAD analysis	(94)
DLLME/SULLE	Phenolics/plums (*Prunus domestica* L.)	**DLLME**: 10 mg dry leaves into Eppendorf tube + 1 mL extraction medium; 200 μL EtAc + 100 μL ACN fast injection; cloudy solution centrifugation; injection (30 s); rest 1 min, 5 min 12,000 × g centrifugation; 100 μL top layer evaporated (N_2_ stream); 50 μL mobile phase resuspension, US (5 min); 20 μL HPLC analysis. **SULLE**: 10 mg dry leaves + 200 μL water + 400 μL ACN; gentle shaking (30 s); 200 μL sugar solution rapidly injected, 1 min vortex; phase separation centrifugation (5 min 12,000 × g); 250 μL top layer dried (N_2_ stream); 50 μL mobile phase resuspension, US (5 min); 20 μL HPLC analysis.	(95)
DLLME (several variations)/SULLE	harpagoside, phenolics/*Harpagophytum procumbens* root, food supplements	50 mg ground roots (40 mesh) + 5 mL water (or 10% NaCl, NADES, IL, glucose or 1% β-cyclodextrin and HP-β-cyclodextrin); 30 s gentle shaking, 600 μL EtAc (extraction solvent) + 500 μl ACN (dispersive solvent); 30 s vortex, 2 min rest (10 min US for UA-DLLME); 4 min 1,500 rpm centrifugation; recover 350 μL top layer; dry (N_2_ stream); 50 μL resuspension (7% ACN, v/v, 3% acetic acid), 20 μL HPLC analysis.	(96)
DLLME	Phytosterols/functional foods, medicinal herbs	Sample (2 mL) + 4 mL water (5 mL glass centrifuge tube); fast mixture injection - 250 μL EtOH (dispersant) + 70 μL bromocyclohexane (extractant); vortex (10 s), US (2 min, 40 KHz); centrifugation (2.5 min, 13,457 × g); sedimented phase transferred to another vial, dry (N_2_ stream); add 120 μL CSR + 50 μL CMPI + 50 μL DMAP ACN; sealed vial radiated (750 W microwave reactor, 5 min, 60°C); dilute resulting solution with 4.0 mL water (5 mL centrifuge tube); add 70 μL bromobenzene (extractant) + 220 μL ACN (dispersant); vortexed (20 s), US (2 min); centrifugation (2.5 min, 13,457 × g); recover bromobenzene (bottom phase); UHPLC-MS/MS analysis/0.005–0.015 ng mL^−1^	(97)
DLLME	Tocopherol/bovine milk	1.0 mL milk sample + 9.0 mL EtOH (containing ascorbic acid, 5 g/L) heating (78°C, 30 min, 10 min intervals shake); ice-cooling, centrifugation (5 min, 4,500 rpm); 1.0 mL supernatant + 200 μL chloroform rapidly injected into 5 mL ultrapure water; cloudy solution centrifuged (5 min, 4,500 rpm); organic phase centrifuged again (10 min, 13,500 rpm), HPLC-PDA analysis/0.01 μg mL^−1^	(98)
DLLME	Flavonols, organosulfurs, inulin/garlic, foods	600 μL chloroform (extraction solvent) + 1 mL ACN (dispersive solvent) injected into sample solution; centrifugation (3 min, 2,000 rpm); chloroform phase dried (N_2_ stream); MeOH reconstitution (500 μL); HPLC-DAD analysis/0.14–2.15 μg mL^−1^	(99, 100)
DLLME	Melatonin and trans-resveratrol/Wine	2 mL centrifuged sample + 7 mL ultrapure water; 1.500 μL ACN (disperser) + 300 μL chloroform (extracting solvent) + 1.500 mg NaCl (ionic strength); mix 1 min; 5 min centrifugation; evaporate the organic phase (N_2_ steam); re-dissolution (150 μL phosphate buffer (40 mM), pH 3/ACN (80/20, v/v); HPLC-FLD analysis/0.07, 7.68 ng mL^−1^	(101)
In-syringe DLLME	Caffeine/coffee	1,500 μL sample + (1,225 μL MeOH + 225 μL DCM extraction); 20 s stirring, repeat; extract water dilution, filtration, HPLC-UV analysis/0.46 μg mL^−1^	(102)
DLLME	Caffeine/tea; energy drinks	2 mL sample + 8 mL deionized water; 1 min shaking; pH adjusted to 3; + 1.5 g NaCl; mix vigorously; + 450 μL EtOH (disperser) + 80 μL 1-octanol (extraction solvent); 1 min mixing; 6,000 rpm (5 min) centrifugation; 20 μL upper phase HPLC-UV analysis/0.9 ng mL^−1^	(103)
DES-HS-SDME	Terpenes/spices	50 mg sample (20 mL HS vial); 10 μL-GC microsyringe containing DES introduced in the HS of the sample vial: DES pushed down the microsyringe to form 1.5 μL drop at the needle tip; incubation (80°C, 90 min); DES drop withdrawn into the microsyringe, disposed in 250 μL insert and weighed; GC-MS analysis/0.47–86.40 μg g^−1^	(104)
M-ILs-SDME	Ascorbic acid (AA)/orange juice	8 mg M-ILs dissolved in a single EtOH droplet (1.0 μL); place on surface of 2 mL phosphate buffer (0.10 M, pH 6.0) containing AA (1.50– 40.0 nM); mixture gently stirred (15 min); AA extracted into M-ILs phase small volume; M-ILs-rich phase collected (strong magnet out the wall of the solution-containing tube, supernatant decanted). M-ILs phase EtOH dilution (3 μL); transfer onto the TiO2-NPs/CPE surface; 2 μL Nafion casted on the electrode; solvent evaporation (RT)/0.43 nM	(105)
IL-UAE	Liquiritin, liquiritin apioside, isoliquiritin, isoliquiritin apioside, glycyrrhizic acid/licorice	1.0 g licorice powder (pulverized, 30 mesh sieves) + 10 mL ILs (several mixtures); US extraction; centrifugation; filtration; HPLC analysis/0.002-0.067 μg mL^−1^	(106)
USAEME	Bioactive compounds/Saffron	79.6 mg saffron sample; 1.1 mL H_2_O (extraction solvent); 18.6 min sonication 62.7 μL chloroform (pre-concentration solvent); RP-HPLC-DAD analysis	(107)
HF-LPME	Quercetin/tomato, onion	1 g crushed, dried sample (6 h, 60°C); 10 mL 25% HCl (80°C, water 30 min, separately); filtration; dilution to 100 mL double distilled deionized water; 10 mL sample (pH 7.5) + 25 μL CTAB + 1-octanol; 30 min 900 rpm stirring, RT/0.1 ng mL^−1^	(108)
HF-LPME	Hesperidin, honokiol, shikonin, magnolol, emodin, β,β′-dimethylacrylshikonin/TCM	Hollow-fiber segment first immersed in organic solvent to fill the solvent in the fiber lumen and wall pore; then the fiber was again immersed into NaCl solution to cover a thin salt membrane on the fiber wall pore filling organic solvent/0.6–12 ng mL^−1^	(109)
DES-HF-LPME	Caffeic acid, cinnamic and p-hydroxycinnamic acids, ferulic acid/ TCM	0.7 mL sample diluted to 7 mL (NaCl 20%, w/v), pH 2 (HCl); DES-immersed fibers (15 min) lumens filled with the 85% DES (MeOH) submerged; 40 min extraction (800 rpm stirring, 55°C); recover the analyte-enriched extractant from fibers lumen; flush lumens with 20 μL MeOH; combine fractions; 30 s vortex; 20 μL HPLC analysis/0.1-03 ng mL^−1^	(110)
OIS-LPME	Alkaloids/TCM	9 mL sample (pH 9) + pentanol/octanol (6:4, v/v) + NaCl (20% w/v) immobilized on permutite surface to form oil-in-salt double membranes; 30 min extraction (25°C, 500 rpm stirring); 50 μL MeOH permutite elution (30 s shaking); 20 μL HPLC analysis/0.1 ng mL^−1^	(111)
BT-OIS-LPME	Magnolol, honokiol/TCM	5 mL sample solution + conditioned ballpoint tip; 30 min 1,200 rpm extraction (RT); ballpoint tip cavity (enriched acceptor phase) rinsing (20 μL MeOH); 20 μL HPLC analysis/0.4, 0.6 ng mL^−1^	(112)
DES-FSME	Curcuminoids/rhizoma turmeric tea	20 mg sample + 0.8 mL of pH 2 HCl; dilution to 8.0 mL (water); 1,100 rpm stirring, 10 min extraction (40°C); add 70 μL DES, mix; rest 5 min; 6 min deep freezing (-20°C); collect solidified droplets; 1:1 MeOH dilution of melted droplets (RT); HPLC analysis/0.2–1 ng mL^−1^	(113)
FSME	Myricetin, quercetin, isorhamnetin, chrysin, kaempferide/TCM	1.0 g sample; 10 min soaking 40 mL MeOH; 30 min US; 30 min herbal extract reflux with 5 mL 25% HCl; 10 min 3,500 rpm centrifugation; adjust supernatant 50 mL; dilute 20 × 6 mL extract + 40 μL dispersed solution of fibroin/dodecanol, 1,200 rpm stirring, 40 min extraction; rest 5 min; 6 min deep freezing (-20°C); collect solidified droplets; 40 μL MeOH elution of melted droplets (RT); 15 min 10000 rpm centrifugation; 10 μL supernatant HPLC-DAD analysis**/**0.2–1 ng mL^−1^	(114)
FSME	Caffeic, ferulic, *p*- and hydroxycinnamic acids/ TCM	10 mL sample (pH 3) + 70 μL graphene/dodecanol (0.25 mg mL^−1^) dispersion; 30 min extraction (1,000 rpm stirring); rest 5 min; 6 min deep freezing (-20°C); collect solidified droplets; 40 μL MeOH elution of melted droplets (RT); 15 min 10,000 rpm centrifugation; 10 μL supernatant HPLC-DAD analysis/0.1–2 ng mL^−1^	(115)
HF-EKE	Caffeine and GA/coffee	5 g coffee + 3 ml water (different pHs), mixed and compacted into an EK cell (65 mm in length, 30 mm internal diameter); cathodic and anodic HFs dipped in 2-nitrophenyl octyl ether containing 5% di-(2-ethylhexyl) phosphate and 1-octanol, respectively. The two HFs (50 ml extraction solvent—-water with different pHs) placed at the ends of the sample compartment; sample solutions collected every 30 min (Hamilton syringe); US bath extraction with various voltages (5–30 V) and times (0.5–5 h); HPLC-PDA analysis.	(116)

*AA, Ascorbic acid; ACN, acetonitrile; APCI-MS, atmospheric-pressure chemical ionization mass spectrometry; BT-IOS-LPME, Ballpoint tip-protected oil-in-salt liquid-phase microextraction; CMPI, 2-Chloro-1-Methyl Pyridinium Iodide; CSR, 4′-Carboxy-substituted rosamine; DAD, diode-array detection; DCM, dichloromethane; DES, deep eutectic solvents; DLLME- dispersive liquid-liquid microextraction; DMAP, 4-dimethylaminopyridine; EtAc, ethyl acetate; EtOH, ethanol; FA, formic acid; FSME, floating solidification microextraction; GA, gallic acid; GC-MS, gas chromatography-mass spectrometry; HF-EKE, hollow fiber electrokinetic extraction; HF-LPME, hollow-fiber liquid-phase microextraction; HS, headspace; ILs, ionic liquids; IOS-LPME, oil-in-salt liquid-phase microextraction; LC-MS, liquid chromatography mass spectrometry; LE, liquid extraction; LLME, liquid-liquid microextraction; M-SPE, magnetic solid-phase extraction; M-ILs-SDME, magnetic ionic liquids single drop microextraction; ME, microextraction; MeOH, methanol; NADES, natural deep eutectic solvents; OS, organic solvents; PLE, pressurized liquid extraction; SHS, Switchable-hydrophilicity solvent; SULLE, sugaring-out assisted liquid-liquid extraction; TCM, traditional Chinese medicine; THF, Tetrahydrofuran; US, ultrasonication; USAEME, ultrasonic assisted emulsification microextraction; VA, vortex assisted*.

### Solid-Phase Microextraction and Related Formats

SPME is certainly one of the most popular extraction methodologies and despite it has been introduced three decades ago, it continues to be an important reference in what concerns microextraction approaches. Often, its usage in food analysis relates to flavor composition, namely the characterization of the volatiles organic compounds (VOCs) that define the aroma of different fruits, like mangoes (51) and tangerines (52). Many of these VOCs are bioactive compounds with promising properties, as thymol identified in tangerines cultivated in Madeira Island (52). The same authors used a related methodology, needle-trap microextraction (NTME) to identify many bioactive VOCs in lemons also grown in the same geography (56). Unlike SPME, NTME is an exhaustive approach that allows a deeper and broader coverage of the volatile composition of different samples, exhibiting improved detection limits and reduced sampling time and volume (117). Moreover, this format allows sampling and storage in the same device (needle trap) for much longer times than SPME, from days to weeks, depending on the type of the target VOCs (117). This makes NTME suitable for sampling procedures distant from the laboratory facilities where the gas chromatography mass spectrometry (GC-MS) analysis is performed. But in contrast to NTME, SPME has been also used to analyze non-volatile bioactive compounds, as phenolics in juices and wines (54, 55). Related with this, Shou and collaborators have been proposed different approaches merging pipette tip microextraction with SPME (PT-SPME) for the quantification of important bioactive flavonoids and alkaloids in different Traditional Chinese Medicine preparations (53, 57). The comparisons made with conventional extractions approaches LE and SPE, clearly point to the potential of the methodologies proposed, namely in what concerns to the higher analytical performance they allow (Table 1). Phenolic compounds are one of the most widespread groups of bioactive compounds and different SPME approaches have been also used to quantify several of these compounds in wines and fruit juices (54, 55, 118) (Table 2).

### Micro Solid-Phase Extraction and Related Formats

Taking into consideration the success and widespread use of SPE, its miniaturization and downscale is a logical consequence of the adoption of GAC requirements, namely in what concerns the usage of organic solvents, which are greatly reduced in the transition from SPE to μSPE. Moreover, the ease of use, time of extraction, availability, and automatization potential, contribute to the success of μSPE as well as different approaches developed. In the traditional concept of μSPE using a sorbent entailed between two fritzes in a syringe tube, as well as in the PT-SPE, that uses instead a pipette tip, new sorbent particles have been successfully reported, for instance, to extract gallic acid (GA). Also known as 3,4,5-trihydroxybenzoic acid, this is a low molecular triphenolic compound widely present in different plants and able to elicit a myriad of bioactive effects in the human body [reviewed in Choubey et al. (119)]. For this reason, its quantification in foodstuffs is very important and, in this aspect, the microextraction approaches reported by Hao et al. (58) and Arabi et al. (61) are very relevant. Hao et al. (58) developed a novel surface imprinting polymer based on magnetic carbon nanotubes (M-CNTs) to extract GA from pomegranate rinds samples, while Arabi et al. (61) reported the use of pipette tip SPME (PT-SPME) using molecularly imprinted silica monolithics to extract the same phenolic acid from juices. Related to this, Khajeh et al. (116) proposed an electrokinetic approach for the simultaneous extraction of GA and caffeine from coffee which is very promising but needs further improvement to lower the extraction times to acceptable values (50 h to achieve 95% extraction, only 20% target analytes extraction after 5 h). The use of molecular imprints obtained against specific analytes elicits more efficient extractions, as reported for estradiol extraction from milk using a PT-SPE approach (62) (Table 2). Another type of sorbent particle, the mesoporous molecular sieve Santa Barbara Amorphous 15 (SBA-15), has been also used with success in μSPEs formats to extract flavanones from citrus fruits (45) and triterpenoid saponins from TCM (63). MEPS and μSPEed formats are very interesting forms of SPE miniaturization in which the sorbent particles are tightly packed inside a BIN (MEPS) or cartridge (μSPEed) and operated in different formats, including hand-held automatic syringes able to control the solvent flow. This allows pressure-driven extractions which are considerably more efficient than conventional SPEs [reviewed in Pereira et al. (120)]. The more recent μSPEed approach uses even lower submicron sorbent particles (3 μm) and a controlled pressure-driven one-way valve that forces the sample through the sorbent particles in a single direction. Overall, these updates to MEPS configuration make μSPEed even more powerful for a broad range of applications, preferentially involving samples with low viscosity to avoid clogging the sorbent containers (120). For this reason, most of the MEPS and μSPEed applications reported so far involve liquid samples or solid samples that suffer a previous solvent extraction or dilution to decrease viscosity. Among the most recent and notable MEPS and μSPEed applications reported in the literature in food analysis, we can find the extraction of phenolic acids in tea (67) and baby food (66) and furanic derivatives in sugarcane honey (Tables 1, 2).

### Microextraction Formats Involving the Dispersion of the Sorbent

A popular approach to microextraction involves the blending of the sorbent with the sample matrix to facilitate the retention of the target analytes in the sorbent particles. This is the extraction principle in MSPD, dispersive SPE (dSPE) and related formats, which then diverge in the form as the sorbent particles and retained analytes are recovered. Often this is achieved by packing in columns, or simply in SPE tubes, to which vacuum is applied, or by centrifugation, followed by the target analytes elution in a suitable elution solvent. In this type of extractions applied to the extraction of bioactive compounds in food lies, the miniaturization to lower the different reagent and particles requirements seems inevitable, as reported to the extractions of phenolics from plant extracts (72) and juices (68). The use of magnetic nanoparticles in the extraction process constitute another important improvement because a simple magnet can be used to isolate the sorbent particles and retain analytes by decantation. This approach has been used to extract different flavonoids from tea and wines (60) and it is being incorporated in other formats to simplify the experimental design of the extraction process, but also the efficiency and selectivity of the methodology. This was achieved, for instance, by coupling magnetic particles with molecular imprinting, as reported in the extraction of different phenolic compounds from fruits and juices (58, 59). But eventually, the major innovation in microextraction of bioactive compounds in food analysis is the use of new and more efficient sorbents, allowing greater retention capacities, as a 2D-ultrathin Ni/Co-NO_3_ layered double hydroxide nanosheet (46) or graphene nanoplatelets (71) to extract phenolic acids from fruit juices and plant preparations. Also, carbon nanofibers (CNFs) were reported to extract phytohormones and ginsenosides (69).

### Microextraction Formats Using Liquid Sorbents

In simple terms, conventional LLE involves the transference (extraction) of analytes present in a liquid sample to an organic and immiscible solvent to which the analytes have more affinity. This usually involves agitation (by mixing, vortexing, etc.) to facilitate the extraction, followed by centrifugation to separate and recover the phase containing the extracted analytes. Often, LLE uses large volumes of organic solvents and consequently generate a large volume of toxic wastes difficult to manage. The miniaturization of LLE contributes to mitigating these problems and several improvements at this level have been proposed. Amini and Hashemi (81), for instance, proposed an LLME approach to extract caffeine from tea and coffee using considerably fewer volumes of organic solvents than conventional LLE. In turn, Shalash et al. (82) reported an LLME using vortex to facilitate the extraction of phenolics from honey, ice tea, and coffee drinks. In the dispersive LLE (DLLE), the organic solvent usually forms micelles in the liquid phase containing the target analytes that increase considerably the extraction efficiency and similarly to LLME, several DLLME approaches have been reported in the literature to extract bioactive compounds from foods, like phenolics (95), vitamin E (98), phytosterols (97) or flavonols, organosulfurs and inulin (99, 100). Related to DLLME, single-drop microextraction (SDME) seems to be also a very efficient extraction procedure, allowing a great preconcentration factor as the volume of the extraction solvent is limited to a single drop. In the original instrumental configuration of SDME, the drop of the extracting (immiscible) solvent is controlled by a microsyringe (or pipette) that loads and withdraw the drop from the solution. This format is not easy to automatize and is often performed with non-commercial devices. However, Jahromi et al. (105) proposed an SDME using magnetic ILs (M-ILs), dispersing the single drop of the solvent containing the M-ILs in the sample solution and recovering it back using a magnet. Using this approach, the authors reported a methodology with a LOD of 0.43 nM for the analysis of AA in orange juice. Liquid-phase microextraction is another interesting approach involving microextraction in liquid phases. The HF-LPME is eventually the most popular approach of this type of microextraction, but recent reports propose other type of containers in which the microextraction of the target analytes occurs, as well as the use of DES. Gao et al. (111), for instance, immobilized the extraction solvent mixtures on a permutite surface to form oil-in-salt double membranes to extract active alkaloids from a TCM powder. In turn, Wang et al. (112) used a protected ballpoint tip to perform a similar oil-in-salt microextraction (IOS-LPME) of the phenolic lignans magnolol and honokiol, that constitute the main primary active ingredients of a TCM. Finally, Zhang et al. (110) reported deep eutectic solvent-based HF-LPME to extract cinnamic acid derivatives in TCM. Floating solidification microextraction (FSME) is an elegant variation of LPME involving the solidification of the extracting phase. Upon the extraction procedure, the enriched phase containing the extracted analytes is fast frozen and easily separated from solution by decantation. Then it is melted at RT and the target analytes re-extracted with MeOH and recovered by centrifugation. This approach has been successfully used to extract important bioactive compounds from different food matrices (Table 2).

### Microextraction Approaches Using Ionic Liquids

The use of ILs in microextraction holds great potential due to their unique “green” properties including thermal stability, re-usage, high reaction efficiency and selectivity at room temperature and ability to dissolve both organic and inorganic compounds (121). For these reasons, ILs are being used in a growing number of applications, like the one referred above using magnetic ILs to extract AA (105). But the future of ILs belongs to the DES, a less toxic and even more ecofriendly subclass of ILs that are easier and cheaper to obtain (122, 123). DES provide a network of hydrogen bond acceptor (HBA) and hydrogen bond donor (HBD) that favor the dissolution process of target analytes (122) and their utility in the microextraction process is not restricted to their use as extraction solvent. DES are also useful to modify and improve the extraction efficiency and selectivity of other sorbents like nanoparticles and silica; to facilitate the dissolution or digestion of solid samples; and to act as elution solvent (123, 124). On top of this, there are natural occurring DES (NADES), that are even more interesting and promising in microextraction, particularly as substitutes of the conventional organic solvents. Bakirtzi et al. (93), for instance, assayed several NADES and showed that they were non-toxic, renewable, and exceptionally efficient solvents for polyphenol recovery from medicinal plants. This higher efficiency is very important because there are many compounds present in plant composition, that despite their important bioactivities, are present in so low concentrations that they remain unidentified (125). Phenolics are certainly one of such family of compounds and for this most of the reports in the literature using DES and different microextraction approaches applied to food matrices have phenolic compounds as target analytes (86, 89, 90, 93, 94, 104, 125). In this context, the work reported by Majidi and Hadjmohammadi (48, 70) combining magnetic nanoparticles and DES in a dSPE format to extract morin, quercetin and kaempferol from different foods is particularly inspiring for other demanding applications envisaging target analytes in very low concentrations in the respective samples (Table 1). Further details of the experimental conditions used in this, and other reports involving DES are available in Tables 1, 2.

### Other Microextraction Approaches

In this section were included microextraction formats involving different mechanisms of retention of target analytes. This is the case of the salting-out mechanism behind the popular QuEChERS extraction. In principle, this would not be considered a microextraction approach considering the different salt and solvent volumes required. However its miniaturization, μQuEChERS, only requiring a fraction of the original recipe of QuEChERS, has been successfully optimized for the extraction and simultaneous determination by UHPL-UV analysis of 12 polyphenols in different baby food samples (50). Similarly, it is relevant to point recent salting-out liquid-liquid extraction (SALLE) approaches able achieve great improvements in the extraction and subsequent analysis of important bioactive compounds. This is the notable examples of the SALLE approach proposed by Park and Jung (79) to extract isoflavones from soy milk, which is 100 times faster than the conventional methodologies used with the same objective. Additional examples to extract naringenin from juices (78) or matrine alkaloids from TCM (80) are described in more detail in Table 1. The use of electric potential in sample microextraction is another format that has been reported for the analysis of bioactive compounds in food. Khajeh et al. (116) proposed a hollow fiber-electrokinetic extraction approach (HF-EKE) for the simultaneous extraction of GA and caffeine from coffee. This is a simple and promising approach but still requires further improvement to lower the extraction times to acceptable values (50 h to achieve 95% extraction, only 20% target analytes extraction after 5 h).

## Final Remarks and Outlook for Future Formats and Applications

In this review, the most recent and innovative microextraction approaches used to extract bioactive compounds from food samples have been presented and their potential and contribution to the whole analytical layout discussed. The major challenges many innovative microextraction approaches must face include their broad application in terms of suitable samples and target analytes, but mainly in terms of potential for their production and distribution across the scientific community. Beyond the analytical aspects, a successful microextraction application needs to be consistently produced and distributed at a commercial level, allowing its usage across different labs and comparison of the results obtained. In this context, the adoption of DES to substitute many conventional organic solvents holds great potential because can be readily used with the sampling and microextraction formats available, not requiring the adoption of new methodologies or acquisition of laboratory materials or instruments and will certainly improve the analytical performance of the currently used methodologies. In contrast, microextraction approaches involving custom sorbents, like MIPS, CNTs and CNFs, despite the superior analytical performance they may allow, will be adopted only if they become commercially available. In turn, MNPs still require additional studies involving their regeneration and durability without affecting the performance of the extraction process. Beyond the type and sorbents feasibility, the microextraction format is also an important aspect to consider, namely the simplicity of the operation mode and the possibility for and easier migration from manual to automatic modes. In this point, miniaturized SPE formats, like μSPEed, will continue to benefit from using traditional formats, but their potential can certainly be further enhanced with the adoption of even more efficient sorbent particles. Eventually, the adoption of DES in the μSPEs formats will make microextraction even more efficient, boosting analytical performance to fulfill the challenges that food analysis involves.

## Author Contributions

JP, NC, and JC contributed to the planning of the review. JP, NC, PP-F, and JC wrote sections of the manuscript. JP edited the manuscript. All authors contributed to manuscript revision, read, and approved the submitted version.

## Funding

This work was supported by FCT-Fundação para a Ciência e a Tecnologia through the CQM Base Fund – UIDB/00674/2020, and Programmatic Fund – UIDP/00674/2020, and by ARDITI-Agência Regional para o Desenvolvimento da Investigação Tecnologia e Inovação, through the project M1420-01-0145-FEDER-000005 – Centro de Química da Madeira – CQM+ (Madeira 14-20 Program). JP was supported by a Post-Doctoral fellowship given by ARDITI (Project M1420 – 09-5369-FSE-000001) and Priscilla Porto-Figueira was supported by FCT (Ph.D. fellowship SFRH/BD/129630/2017).

## Conflict of Interest

The authors declare that the research was conducted in the absence of any commercial or financial relationships that could be construed as a potential conflict of interest.

## Publisher's Note

All claims expressed in this article are solely those of the authors and do not necessarily represent those of their affiliated organizations, or those of the publisher, the editors and the reviewers. Any product that may be evaluated in this article, or claim that may be made by its manufacturer, is not guaranteed or endorsed by the publisher.

## Glossary

AA: Ascorbic acid
ACN: acetonitrile
APCI-MS: atmospheric-pressure chemical ionization mass spectrometry
BIN: barrel insert needle
[Bmin]BF_4_: 1-butyl-3-methylimidazolium tetrafluoroborate
BT-IOS-LPME: Ballpoint tip-protected oil-in-salt liquid-phase microextraction
CAD: charged aerosol detector
CNFs: carbon nanofibers
CMPI: 2-Chloro-1-Methyl Pyridinium Iodide
CNTs: carbon nanotubes
CSR: 4′-Carboxy-substituted rosamine
DAD: diode-array detection
DCM: dichloromethane
DES: deep eutectic solvents
DLLME: dispersive liquid-liquid microextraction
DMAP: 4-dimethylaminopyridine
dSPE: dispersive solid-phase extraction
DVB: divinylbenzene
ECD: electrochemical detection
EPT-SPME: effervescent pipette tip solid phase microextraction
EtAc: ethyl acetate
EtOH: ethanol
FA: formic acid
FGO-TD-PTFE: functional graphene oxide thermal desorption poly(tetrafluoroethylene)
FLD: fluorescence detector
FSME: floating solidification microextraction
GA: gallic acid
GAC: Green Analytical Chemistry
GC-MS: gas chromatography-mass spectrometry
HAc: acetic acid
HF-EKE: hollow fiber electrokinetic extraction
HF-LPME: hollow-fiber liquid-phase microextraction
HLB: hydrophilic-lipophilic-balanced
HS: headspace
ILs: ionic liquids
IOS-LPME: oil-in-salt liquid-phase microextraction
LC-MS: liquid chromatography mass spectrometry
LDH: layered double hydroxide
LDLs: low-density lipoproteins
LE: liquid extraction
LLME: liquid-liquid microextraction
M-ILs-SDME: magnetic ionic liquids single drop microextraction
MIP: molecular imprinted polymer
MIOMS-ir: molecularly imprinted ordered mesoporous silica imprint-removed silica
MISM: molecularly imprinted silica monolithic
ME: microextraction
MeOH: methanol
MEPS: microextraction by packed sorbent
MMF: multiple monolithic fiber
MNPs: magnetic nanoparticles
MOF: Metal–organic framework
MSPDM: matrix solid-phase dispersion microextraction
MWCNTs: Multi-walled CNTs
M-SPE: magnetic solid-phase extraction
NADES: natural deep eutectic solvents
NCS: nanoconfined solvent
NPs: nanoparticles
NTD: needle trap device
NTME: needle-trap microextraction
OS: organic solvents
PDA: photodiode array
PDMS: polydimethylsiloxane
PLE: pressurized liquid extraction
PT: pipette tip
PS/DVB-RP: reverse phase polystyrene-divinylbenzene sorbent
RAMIPs: restricted access molecularly imprinted polymers
RT: room temperature
SALLE: salting-out liquid-liquid extraction
SBA-15: Santa Barbara Amorphous 15 molecular sieve
SHS: Switchable-hydrophilicity solvent
SE: solvent extraction
SPME: solid-phase microextraction
SULLE: sugaring-out assisted liquid-liquid extraction
TCM: traditional Chinese medicine
THF: Tetrahydrofuran
US: ultrasonication
UHPLC: ultrahigh performance liquid chromatography
USAEME: ultrasonic assisted emulsification microextraction
UV: ultraviolet analysis
VA: vortex assisted
VOCs: volatile organic compounds
μSPE: micro solid-phase extraction.
